# Epidemiological pattern of trauma patients based on the mechanisms of trauma: trends of a regional trauma center in Midwest of Iran

**DOI:** 10.1186/s12873-022-00756-9

**Published:** 2022-12-26

**Authors:** Ghodratollah Roshanaei, Sahar Khoshravesh, Sajjad Abdolmaleki, Tayebeh Bathaei, Mahnaz Farzian, Mohammadreza Saatian

**Affiliations:** 1 Department of Biostatistics, School of Public Health, Modeling of Noncommunicable Diseases Research Center, Hamadan University of Medical Sciences, Hamadan, Iran; 2 Social Determinants of Health Research Center, Hamadan University of Medical Sciences, Hamadan, Iran; 3 Department of Neurosurgery, Hamadan University of Medical Sciences, Hamadan, Iran; 4 Department of Operating Room, School of Para Medicine, Hamadan University of Medical Sciences, Hamadan, Iran; 5 Clinical Supervisor, Be’sat hospital, Hamadan, Iran; 6 School of Medicine, Hamadan University of Medical Sciences, Shaheed Fahmideh Ave, Hamadan, Islamic Republic of Iran

**Keywords:** Epidemiological pattern, Trauma, Multinomial Logistic Regression, Iran

## Abstract

**Introduction:**

Trauma is one of the important issues in public health because it is responsible for 90% of mortality in Low and Middle-Income Countries (LIMCs). The present study aimed to determine the epidemiological pattern of trauma patients in a regional trauma center in the Midwest of Iran from 2014 to 2020.

**Methods:**

This study was a retrospective study that was performed on 29,804 trauma patients admitted to Be′sat Hospital in Hamadan from January 2014 to December 2020. Data was collected using Health Information Management (HIM) Center of the Be′sat Hospital. For investigating the relationship of the characteristics of trauma patients and the mechanisms of trauma, Multiple Multinomial Logistic Regression (MMNLR) model was used. All statistical analyses were performed using the IBM SPSS Statistics version 24.

**Results:**

The mean age of all patients was 35.4 (SD = 21.9) years. Most of them were men (71.7%). The most common mechanism of trauma was road traffic accidents (RTAs) (39.6%) followed by falls (30.2%), other (19.7%), violence (6.2%), and burn (4.4%). 1.5% of the trauma patients expired. The results of multiple multinomial logistic regression indicated that significant affected factor on odds referring because of *RTAs* compared to other mechanism were: season and hospital length of stay (LOS); in *falls* and *violence*: age, sex, season, and LOS; and in *burn*: age, sex, season, evening time, and LOS (*p* < 0.05).

**Conclusion:**

Based on the investigation of 29,804 trauma patients, in Iran as a developing country, RTAs and falls were two common mechanisms of trauma. It seems that as a short-term plan, it is possible to focus on road safety, to improve the quality of vehicles, to hold training courses for drivers. Also, as a long-term goal, considering that the elderly population in Iran is increasing, it is necessary to pay attention to fall reduction programs.

## Introduction

Trauma is the leading cause of death, hospitalization, and disability in the world [1, 2]. Trauma causes 10% of global mortality [3] and it is responsible for 90% of mortality in Low and Middle-Income Countries (LIMCs) [4]. Trauma also decreases the disability adjusted life years (DALY) in different communities [5]. Today, trauma is one of the important issues in public health that should be given more attention in the health care system, because not only thousands of deaths and millions of injuries occur due to various trauma annually [6], but also it imposes direct and indirect socio-economic costs on the health care system of communities and individuals [7]. These costs include treatment costs, reduced productivity, stopping activities, and loss of family income at the time of hospitalization [8].

Iran as a middle-income developing country faces a high mortality and morbidity rate due to trauma [9]. In Iran, after cardiovascular disease as the first cause of death, road traffic accidents (RTAs) are the second cause of mortality [10], the first cause of years of life lost (YLL), and the common cause of injury [11, 12]. In Iran, the rate of mortality related to RTAs is higher Eastern Mediterranean region (EMR) [13], with 30 deaths per 100,000 people in Iran Mediterranean [2] compared with 14 deaths per 100,000 people in the EMR [11]. Unfortunately, it has been increasing during recent years [13].

Trauma can be classified according to the mechanism (RTAs, fall, violence, etc.), the area (head, limbs, etc.), and the features of the causative factor (penetrating, blunt, or barotrauma) [14]. The mechanisms of trauma have been reported very diverse in different countries. For example; the results of a study in Italy indicated that RTAs, especially involving motorcycles, were the most common cause of injury.

(69.5%) and followed by falls (12.3%) [15]. The study of Pogorzelski et al., in Brazilian people pointed out that 60.4% of trauma patients suffered RTAs (52% motorcycle), and 31.2% were violence victims [16]. A nationwide review of seven million emergency department admissions in Iran showed that the most common mechanism of trauma was RTAs (31.0%) and followed by hit (28.2%) and fall (10.1%) [17], while the most common mechanism of trauma in Korea was the blunt injury (90.8%) [18], and in India, it was related to a fall (75.6%) [19]. It sounds that special strategies must be designed and implemented to control and prevent various the mechanisms of trauma in each community.

Considering the high prevalence of trauma, high-rate mortality, and severe complications due to trauma in Iran, it seems that the use of preventive methods in the field of trauma is effective when accurate information and statistics about the incidence of various mechanisms of trauma and its consequences are available in the community. Determining the common mechanisms of trauma in this study can be helped to develop the treatment protocols for identifying at-risk groups and their special care. This may improve the quality of services provided to trauma patients. Although nature trauma is well known, the mechanisms of trauma are less known especially in developing countries where few studies have been conducted on the epidemiology of trauma and its mechanisms [20]. To our knowledge, there are no published data about the epidemiological pattern of trauma patients based on common mechanisms of trauma. Therefore, the current study aimed to determine the epidemiological pattern of trauma patients based on common mechanisms of trauma in a regional trauma center, Be′sat Hospital, in Hamadan from January 2014 to December 2020.

## Methods

### Study design

This study was a retrospective study (from January 2014 to December 2020.).

### Setting

Different wards of Be′sat Hospital in Hamadan, Iran. Be′sat Hospital is a regional trauma center in Hamadan, Midwest of Iran.

### Participants

Trauma patients admitted to different wards of Be′sat Hospital.

### Variables

Characteristics of trauma patients including age, sex, season, time of week, referral time, mechanisms of trauma, history of hospitalization, hospital length of stay (LOS), surgery, and final status of patients was investigated.

### Data sources/measurement

In this study, data source was Health Information Management (HIM) Center of the Be′sat Hospital.

### Study size

Twenty-nine thousand eight hundred four trauma patients who had been admitted to different wards of Be′sat Hospital.

### Quantitative and Qualitative variables

Quantitative variables were presented as mean and SD. Qualitative variables were expressed as frequency and percentages.

### Statistical methods

The mechanisms of trauma were considered as the dependent outcome variable (including five mechanisms such as accident, fall, violence, burn and other mechanisms). It is noteworthy that in this study, “other” in the mechanism of trauma included drowning, electrocution, suicide, homicide, poisoning, animal attacks, bites, and playing (sports) trauma. For investigating the relationship of the demographic and the mechanisms of trauma, Multiple Multinomial Logistic Regression (MMNLR) model was used. In MMLR model, “other” in the mechanisms of trauma was considered as reference group and the effect of each risk factor was measured on each mechanism of trauma compared to other. The results of model fitting were presented as Odds Ratios (OR) and their 95% confidence intervals. *P* value < 0.05 considered to be as significant. All statistical analyses were performed using the IBM SPSS Statistics version 24.

## Results

### Participants

Twenty-nine thousand eight hundred four trauma patients who had been admitted to different wards of Be’sat Hospital from January 2014 to December 2020.

### Descriptive data

The mean age of all patients was 35.4 (SD = 21.9) years. The mean age of males and females was 32.2 (SD = 19.9) and 40.9 (SD = 21.9), respectively. Most of the trauma patients were men (71.7%). The most referral season was summer (32.1%). About half of the referrals were recorded in the evening (46.4%). 70% of patients were hospitalized with 3 or less days. 95.8% of patients were discharged from the hospital and 1.5% of the trauma patients expired (Table 1).

**Table 1:** Characteristics of patients (*n* = 29,804)

Characteristics	Frequency	
	Number	Percent ((%)
**Age (Year)** Mean ± SD (35.4 ± 21.9)		
≤ 18	7093	23.8
> 18	22,711	76.2
**Sex**		
Male	21,395	71.7
Female	8409	28.3
**Seasons**		
Spring	7562	25.4
Summer	9589	32.1
Autumn	7855	26.4
Winter	4798	16.1
**Time of week**		
Weekends	8995	30.2
Weekdays	20,809	69.8
**Referral time**		
Morning	3394	11.4
Evening	13,824	46.4
Night	12,586	42.2
**Mechanism of trauma**		
Road Traffic Accident	11,790	39.6
Falls	9009	30.2
Violence	1836	6.2
Burn	1308	4.4
Other	5861	19.7
**History of hospitalization**		
Yes	6938	23.3
No	22,866	76.7
**Hospital length of stay**		
≤ 3	20,843	70.0
> 3	8961	30.0
**Surgery**		
Yes	24,036	80.6
No	5768	19.4
**The final status of patients**		
Discharged	28,529	95.8
Escaped	827	2.7
Expired	448	1.5

**Fig. 1:**
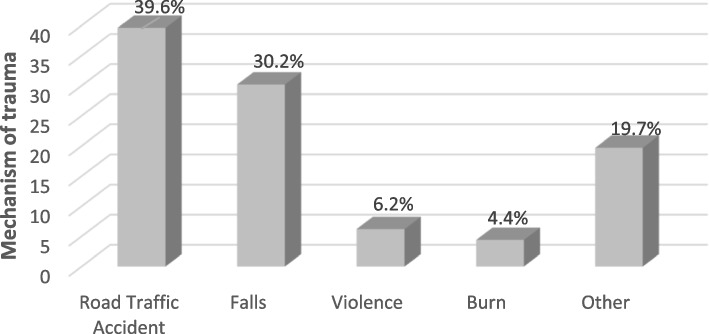
Mechanism of trauma in trauma patients

The most common the mechanism of trauma was RTAs (39.6%) followed by fall (30.2%) (Fig. 1).

### Outcome data

The results of multiple multinomial logistic regression (MMLR) model indicated that significant affected factor on the odds of referring because of *RTAs* compared to other were: spring and > 3 days hospital length of stay (LOS); odds referring because of *falls* compared to other in following variables were significant: age of > 18 years, being males, spring, summer, autumn, > 3 days LOS; odds referring because of in *violence* compared to other: age of > 18 years, being males, spring, autumn, and > 3 days LOS; and odds referring because of in *burn* compared to other: age of > 18 years, being males, spring, summer, autumn, evening time, and > 3 days LOS were statistically significant (Table 2).

**Table 2:** The results of multiple multinomial logistic regression (MMNLR) model

Variables		Accident Vs. other			Fall Vs. other			Violence Vs. other			Burn Vs. other		
		OR	95% CI	p	OR	95% CI	P	OR	95% CI	p	OR	95% CI	P
**Age (Year)**	≤ 18	1.00			1.00			1.00			1.00		
	> 18	1.01	(0.94–1.09)	0.78	0.90	(0.83–0.97)	**0.01**	1.58	)1.38–1.81)	**< 0.01**	0.52	(0.46–0.60)	**< 0.01**
**Sex**	Male	1.01	(0.93–1.07)	0.89	0.72	(0.67–0.78)	**< 0.01**	2.14	(1.85–2.46)	**< 0.01**	0.79	(0.69–0.90)	**< 0.01**
	Female	1.00			1.00			1.00			1.00		
**Seasons**	Spring	0.81	(0.73–0.90)	**< 0.01**	0.67	(0.60–0.75)	**< 0.01**	0.82	(0.69–0.97)	**0.02**	0.47	(0.39–0.57)	**< 0.01**
	Summer	1.01	(0.91–1.12)	0.77	0.76	(0.69–0.84)	**< 0.01**	0.97	(0.83–1.15)	**0.79**	0.55	(0.46–0.65)	**< 0.01**
	Autumn	1.03	(0.92–1.14)	0.55	0.86	(0.77–0.95)	**0.01**	0.76	(0.64–0.90)	**0.01**	0.70	(0.59–0.84)	**< 0.01**
	Winter	1.00			1.00			1.00			1.00		
**Weekdays**	Weekends	1.00			1.00			1.00			1.00		
	Non-weekends	1.02	(0.95–1.09)	0.58	1.01	(0.94–1.09)	0.68	0.90	(0.81–1.01)	0.09	0.91	(0.80–1.04)	0.21
**Referral time**	Morning	0.96	(0.86–1.07)	0.53	1.05	(0.94–1.17)	0.38	1.03	(0.86–1.23)	0.74	1.14	(0.94–1.38)	0.17
	Evening	0.96	(0.90–1.03)	0.35	1.00	(0.93–1.07)	0.96	1.06	(0.94–1.18)	0.31	0.86	(0.76–0.98)	**0.03**
	Night	1.00			1.00			1.00			1.00		
**History of hospitalization**	Yes	0.97	(0.90–1.05)	0.52	0.99	(0.92–1.07)	0.94	0.95	(0.84–1.08)	0.49	1.02	(0.89–1.18)	0.69
	No	1.00			1.00			1.00			1.00		
**length of stay**	≤ 3	1.00			1.00			1.00			1.00		
	> 3	4.00	(3.69–4.35)	**< 0.01**	2.36	(2.17–2.58)	**< 0.01**	1.38	(1.20–1.59)	**< 0.01**	5.32	(4.66–6.07)	**< 0.01**
**Surgery**	Yes	0.98	(0.90–1.06)	0.65	1.05	(0.97–1.14)	0.21	0.95	(0.84–1.09)	0.52	1.13	(0.96–1.32)	0.13
	No	1.00			1.00			1.00			1.00		
**The final status of patients**	Discharged	0.84	(0.68–1.02)	0.09	0.89	(0.72–1.10)	0.30	0.80	(0.59–1.10)	0.19	1.22	(0.80–1.88)	0.34
	Expired	0.82	(0.59–1.13)	0.24	0.77	(0.54–1.08)	0.14	0.80	(0.47–1.35)	0.42	1.41	(0.75–2.62)	0.28
	Escaped	1.00		1.00			1.00				1.00		

*OR* Odds ratio, *CI* Confidence interval

## Discussion

The current study aimed to determine the epidemiological pattern of trauma patients in a regional trauma center, Be′sat Hospital, in Hamadan from January 2014 to December 2020. The strengths of this study consisted of a large sample size and the ability to examine the epidemiological pattern of common trauma mechanisms separately.

Our results indicated that most trauma patients were > 18 years and males. Other studies have pointed to this [17, 18]. The age of > 18 years was considered as the working-age population [21]. This population is at risk for different trauma. Evidence shows that 35% of the unintentional trauma in this group could be preventable [22]. Also, in this study similar to previous studies, it is expected that according to the nature of the occupational status of men compared to women, men are more susceptible to trauma [19, 23]. Summer is known as “Trauma Season” [24]. In the current study, most of the trauma was also recorded in the summer compared to other seasons, which is consistent with results from other studies [17, 24]. This result could be because of increasing outdoor activities and more desire to travel in our country in summer season similar to many countries.

The most common mechanism of trauma was RTAs. This finding is consistent with previous studies [2, 17, 25]. In Iran, it is estimated that one person dies due to The most common the mechanism of trauma was RTAs every 19 min and a person is injured every 2 min [26]. RTAs occur because of human errors, poor safety, or unsafe vehicles and roads that among these, human errors are the most common cause of RTAs [27]. Some human errors contain a violation of road safety rules, speeding and overtaking illegally, poor driving skills, fatigue, and talking on the phone while driving [27, 28].

In this study, RTAs had a significant association with the season and hospital length of stay. So, the odds of referral due to RTAs compared to other, in patients in spring compared to winter decreased and this decrease was significant. Haji Aghajan et al. reported that the number of RTAs was higher in the summer followed by spring [17]. One of the possible reasons for this contradiction is that due to the geographical conditions and the cold and mountainous nature of Hamadan, the roads of this city are more susceptible to RTAs in winter. Also, the odds of referral to the hospital due to RTAs compared to other was four times higher in patients who were hospitalized for more than 3 days. This is in agreement with similar reports [29, 30]. Kashkooe et al. found that factors such as being male, older age, infection, site of injury (face and thorax), and surgery can be increased the length of hospitalization in trauma patients caused by RTAs [29]. Moreover, the longer length of stay had a significant association with the severity of injury and mortality [31].

In line with the results of our study, in other studies falls were the second cause of trauma [2, 23, 32]. Falls can lead to serious injuries like different fractures and traumatic brain injuries (TBI) [33]. About 70% of people with TBI are children, or young adults [34] that need to be hospitalized [35]. The current study revealed that falls had a significant association with age, sex, season, and hospital length of stay.

Patients with ≤18 years were more likely than patients over 18 years to transfer to the hospital for trauma caused by falls. This finding is documented; For example, a Swedish study of trauma patients under 19 years of age showed that falls were the most common injury among all age groups, except adolescents [35]. Also, Park et al. reported that the most common mechanism of injury at the ages of zero to 9 years was falls which resulted in severe and critical injuries in 3.5% of them [36]. The evidence indicates that the causes of falls in younger children were slipping from the caregiver’s arms, falling off the furniture, bed, or playgrounds [37]. The study of Pandey et al. in Nepal among school-going adolescents demonstrated that the most common cause of injury was fall (56.40%). Severe injuries had a significant association with physical fight or attack, and being bullied [38]. Considering the higher prevalence of fall in patients with ≤18 years in our study, it is important to pay more attention to design the preventive interventions at home and school to reduce fall at these ages.

Females of our study were more likely than males to transfer to the hospital for trauma caused by falls. There are contradictory findings in the literature in this regard. For example; in some studies, females had a higher rate of fall [39–41] and in other studies, males had a higher rate of fall [2, 23]. The reasons for these inconsistent results can be included in different age groups, sex ratio, or occupations of subjects in various studies. For example; in our study, although the number of traumatic men was about 2.5 times of women, the mean age of women was higher than men (40 years vs. 32 years). Evidence indicates that the incidence of falling increases with age in women [42].

According to the definition of WHO about the typology of violence, it has three types: a) *Self-directed violence* (the perpetrator and the victim are the same individual and is included self-abuse and suicide). b) *Interpersonal violence* (violence between individuals, and is included family/intimate partner violence and community violence. Child maltreatment, intimate partner violence, and elder abuse are related to family/intimate partner violence and assault by strangers. Violence related to property crimes and violence in workplaces is related to community violence.

c) *Collective violence* (violence committed by larger groups of individuals and is included social, political, and economic violence) [43]. In this study, self-directed violence was categorized into the “other” subgroup and the interpersonal and collective were categorized into the “violence” subgroup in the cause of trauma. In the current study, violence had a significant association with age, sex, season, and hospital length of stay. Patients over 18 years were more likely than patients with ≤18 years to transfer to the hospital for trauma caused by violence. Mollazehi et al. reported that 92.7% of hospitalized patients because of violence-related injuries in a level 1 trauma center in Qatar were > 18 years old [44]. Also, they found that the most common mechanism of trauma was interpersonal violence (76.7%).

Males were more likely than females to transfer to the hospital for trauma caused by violence. This finding was reported in prior studies [17, 23]. Haji Aghajan et al. found that the male to female ratio for trauma caused by violence was 3.3 and 0.7 for taking place violence in street /public places and home, respectively. It is noteworthy that violence in street / public places mostly involves men and domestic violence involves mainly women [17]. So, it seems that gender-based violence prevention programs could be considered to reduce violence in society.

The odds of referral to the hospital due to violence significantly decreased compared to other of trauma in spring and autumn compared to winter and this decrease was significant. This result is in contrast to the results of a study in Norway. The study pointed out that the highest frequency of violence had occurred in June. Reasons for these contradictory results could be included such as differences in geographical locations, climatic, genetic, and social factors [45].

Based on the latest report of WHO, although burns are preventable, about 180,000 deaths every year are caused by burns that the majority of them occur in low- and middle-income countries [46]. Our study demonstrated that burn had a significant association with age, sex, season, and hospital length of stay.

Women and children are vulnerable to burns [46]. Patients with ≤18 years were more likely than patients over 18 years to transfer to the hospital for trauma caused by burning. In the study of Saberi et al. was reported that almost 50% of patients were less than 16 years and most of them [47]. Given that one of the predictor factors of mortality among burn patients has been reported age ≥ 18 years [48], it sounds that.

There are conflicting results about being at risk of burns based on gender in prior researches. For example; in line with our study, Cutillas et al. found that women were more frequently victims of burns than men [49]. According to the report of WHO, females are particularly vulnerable to burns because of open fire cooking, or inherently unsafe cook stoves. Moreover, self-directed or interpersonal violence are also factors for women’s higher risk of burns [46]. On the contrary our finding, the results of a meta-analysis study in Iran showed that 60% of burn patients were men [47]. As well, Al Laham et al. pointed out that men were more at risk of burns than women [50]. This finding might be because of having more risky jobs and more responsibility for outdoor duties in men than women [50, 51]. Apart from gender, it is necessary to provide education related to prevent burn trauma in communities and to warn about safety tips at homes and at workplaces.

Although, the odds of referral to the hospital due to burn significantly decreased in the evening, Chein reported that the most common hours for occurring burn were 10:00 to 12:00 h and 16:00 to 18:00 h when family members are preparing lunch and dinner [52]. In our study, the odds of referral to the hospital due to burn significantly increased in winter, Lam et al. found that burns have been occurred more in the summer than in other seasons [53]. The cold and mountainous climate of Hamadan and the high cold weather in winter can be one of the important reasons for the increase in burn events in this season. Because people have to use different heating devices in homes and workplaces.

Hospital length of stay (LOS) is considered as an important criterion to evaluate trauma care [54]. Reduction in LOS not only can improve the outcomes in trauma patients but also can decrease hospital mortality and reduce the costs of trauma patients [55]. In our study, odds of referral to the hospital due to RTAs, fall, violence, and burn compared to other causes was significantly higher in trauma patients who were hospitalized for more than 3 days (OR = 4, 2.36, 1.38, and 5.32 times, respectively). So, among the mechanisms of trauma, burn patients had the highest odds of being hospitalized for more than 3 days. This finding has been confirmed by other studies. For example; in the study of Chein et al. was reported that the LOS average for burn patients was 18 days (range = 1–384 days) [52]. Gurbuz and Demir found that the mean of LOS was 14.7 days (range = 1–136 days) for adult burn patients [48]. It seems that given the longer LOS in burn patients compared to other trauma patients, it is important to pay more attention to the design and implementation of programs for reducing LOS in burn patients.

### limitations

Our study had two limitations. First, the mortality rate of trauma patients was based on hospitalization only. Second, we were not able to investigate the Injury Severity Score (ISS). Because this score was not recorded in the Health Information Management (HIM) Center of the studied hospital.

### Generalizability

Large sample size was the strength of this study. Larger sample sizes give more reliable results with greater precision and power.

## Conclusion

Based on the investigation of 29,804 trauma patients, in Iran as a developing country, RTAs and falls were two common mechanisms of trauma. It seems that as a short-term plan, it is possible to focus on road safety, to improve the quality of vehicles, to hold training courses for drivers. Also, as a long-term goal, considering that the elderly population in Iran is increasing, it is necessary to pay attention to fall reduction programs.

## Data Availability

The analyzed dataset in this study is available from the first author on reasonable request.

## Abbreviations

LIMCs: Low and Middle-Income Countries
HIM: Health Information Management
MMNLR: Multiple Multinomial Logistic Regression
RTAs: Road Traffic Accidents
LOS: Hospital Length of Stay
DALY: Disability Adjusted Life Years
YLL: Years of Life Lost
EMR: Eastern Mediterranean region
ISS: Injury Severity Score
